# Stroke from Vasospasm due to Marijuana Use: Can Cannabis Synergistically with Other Medications Trigger Cerebral Vasospasm?

**DOI:** 10.1155/2016/5313795

**Published:** 2016-10-19

**Authors:** Marium Jamil, Atif Zafar, Syed Adeel Faizi, Ifrah Zawar

**Affiliations:** ^1^Dow University of Health Sciences, Karachi, Pakistan; ^2^Cerebrovascular Center, Cleveland Clinic, Cleveland, OH, USA; ^3^Department of Neurology, University of New Mexico, Albuquerque, NM, USA

## Abstract

We present a case of imaging proven cerebral vasospasm causing ischemic stroke in a young patient chronically on buprenorphine-naloxone for heroin remission who started smoking cannabis on a daily basis. With cannabis legalization spreading across the states in the USA, it is important for physicians not only to be aware of cannabis reported association with cerebral vasospasm in some patients but also to be on the lookout for possible interacting medications that can synergistically affect cerebral vessels causing debilitating strokes.

## 1. Introduction 

Ischemic and hemorrhagic strokes have traditionally been associated with several drugs with vasoactive properties [1] such as serotonergics, sympathomimetics (e.g., pseudoephedrine), and chemotherapeutic drugs [1–3]. Illicit drugs including ecstasy, phencyclidine, lysergic acid diethylamide (LSD), cannabis, and heroin have been sporadically reported to be related (if not causative) to stroke [4]. One such commonly used drug is cannabis (street name: marijuana or pot), which is often considered innocuous. A survey conducted by National Institute on Drug Abuse (NIDA) showed that, in 2014, there was a 49.20% lifetime prevalence of illicit drug abuse in the population aged 12 years or older. Cannabis is the most widely used drug in the Unites States with a lifetime prevalence of 44.20% reported in the 2014 survey. The highest lifetime prevalence of cannabis is in relatively younger age group [5]. Several case reports support a causal link between cannabis use and cerebrovascular events [6, 7]. Cannabis has widely been used in the Western societies with an increase in consumption following the recent legalization in several states in the US [8].

Buprenorphine is a partial mu-agonist used in the long term treatment of patients with opioid dependence. At times, a combination of naloxone (an opioid antagonist) is used with buprenorphine for this purpose. Currently, there is no established data to suggest an increase in incidence of cerebrovascular events in patients taking buprenorphine with no other identifiable risk factors. With an increasing push for legalization of medical and edible cannabis across several states, it is imperative that the effects of these drugs are explored and documented, and their causal relationship with stroke is described. We recently treated a young patient with ischemic cerebral stroke who was on a combination of buprenorphine-naloxone for opioid dependence, while he continued to smoke cannabis regularly. We report the first ever case of ischemic stroke secondary to vasospasm in a cannabis user who was also on buprenorphine-naloxone for opioid dependence.

## 2. Case Report 

**Figure 1:**
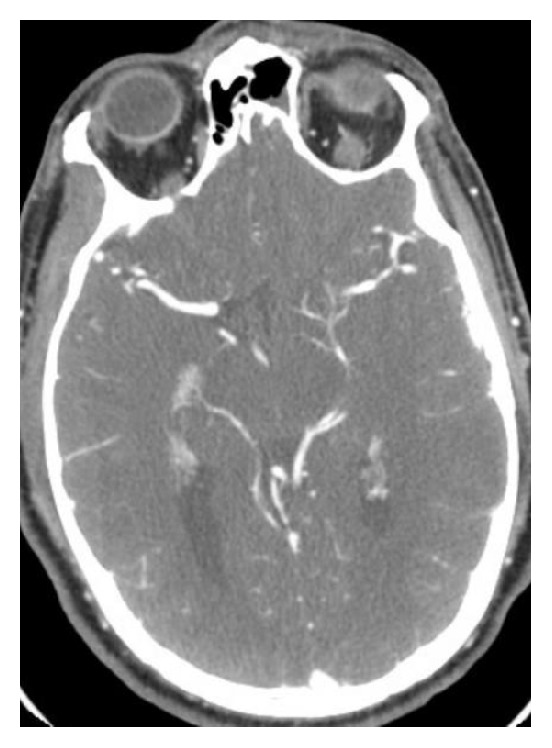
CT angiogram sequence of the left middle cerebral artery at the bifurcation with narrowing.

**Figure 2:**
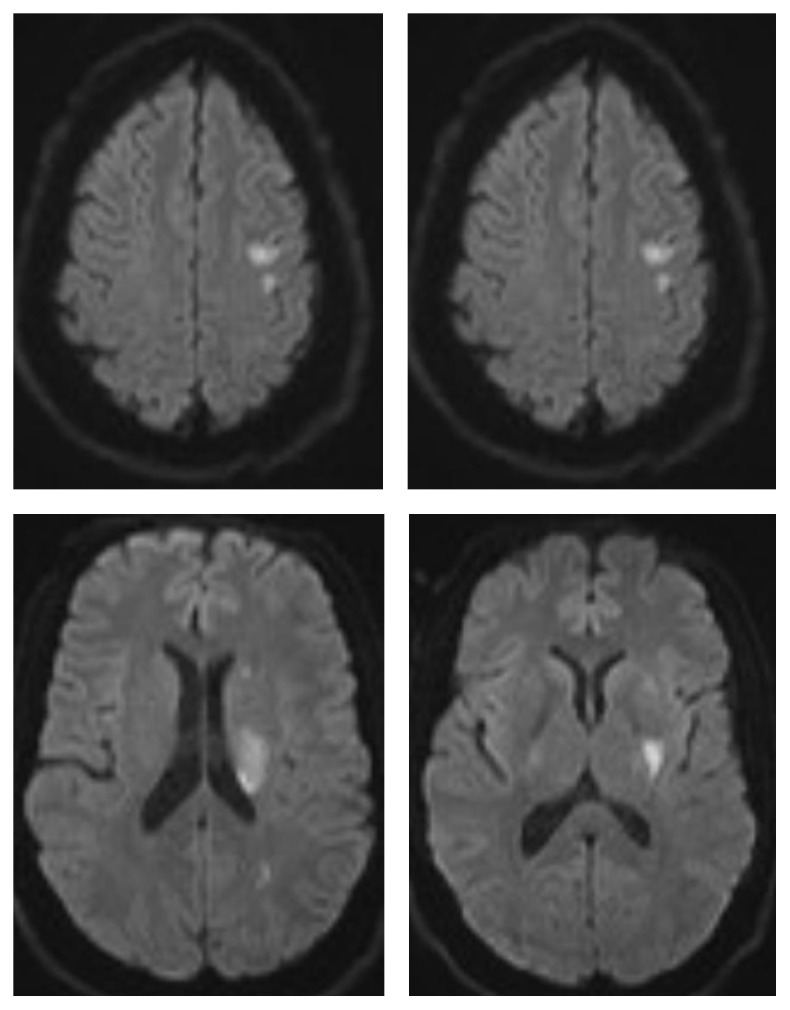
MRI brain DWI sequence demonstrating restriction diffusion in left middle cerebral artery distribution.

**Figure 3:**
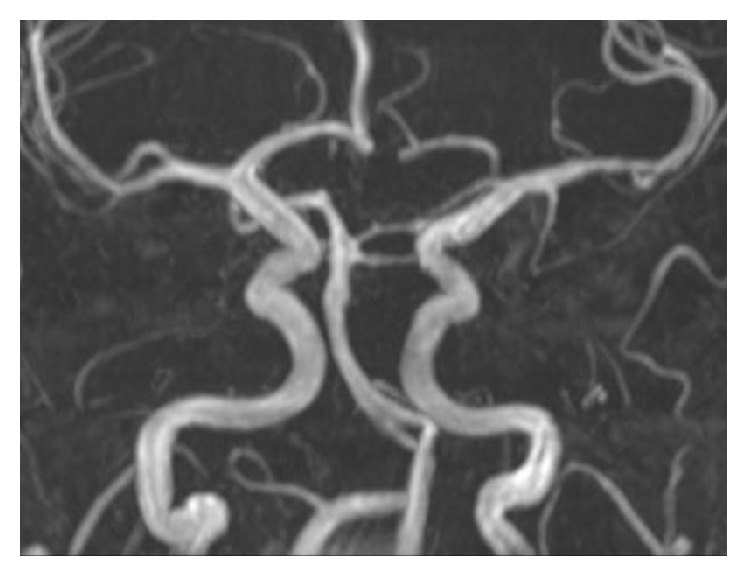
MR angiogram demonstrating patent left MCA. However, if we compare it to the right MCA, there still seems to be some residual stenosis. This MRA was done, a few hours after the initial CT angiogram and at the time patient started to show some fluctuating improvement in his exam.

A 33-year-old male on buprenorphine-naloxone 4-1 mg for heroin dependence presented to an outside facility with an acute onset of right sided weakness and dysarthria approximately 4 hours after initial development of his symptoms. Social history was significant for daily cannabis use and 1 pack per day smoking history of more than 12 years. His last use of heroin was more than 3 years ago. He reported a 3-day history of fever, chills, night sweats, and malaise. Investigations showed leukocytosis (15.5) and a positive urine toxicology screen for tetrahydrocannabinol (THC). CT head showed no acute changes. However, CTA of the head and neck demonstrated a left middle cerebral artery (MCA) narrowing (distal left M1 and proximal M2) (Figure 1). NIHSS at the time of presentation was 5. He was given normal saline followed by Lipitor 80 mg and aspirin 325 mg. He had worsening neurological deficits at the time of his transfer to a comprehensive stroke center (NIHSS 10). Physical exam showed a hemiplegic right upper and lower extremities with dysarthria. An MRI showed patchy diffusion restrictions in the left MCA territory suggestive of acute ischemic stroke as shown in Figure 2. MRA showed partial resolution of left MCA vasospasm (Figure 3). A perfusion scan was also performed and showed symmetrical mean transit and time to peak. Patient showed some improvement and he was admitted to neurocritical unit. The next morning, approximately 12 hours after his first CTA that showed M1 vasospasm, he underwent a diagnostic cerebral angiogram which was completely normal with patent and normal appearing left MCA. Lumbar puncture was performed to work up infectious and autoimmune causes of the stroke, which came back as negative. Transthoracic ECHO (TTE) and Transesophageal ECHO (TEE) were done to evaluate endocarditis, which were also unremarkable. Diagnosis of acute left MCA stroke secondary to vasospasm due to illicit drug use was established. His symptoms resolved over the course of next 48 hours and was discharged with a NIHSS of 0. For secondary prevention, he was discharged on aspirin. Lower dose of buprenorphine-naloxone, weaning off on cannabis use, and smoking cessation were recommended. A 3-week cardiac telemetry monitoring was ordered which came back negative.

## 3. Discussion 

The pharmacokinetic properties of cannabis depend upon the route of administration, whereby smoked cannabis causes its psychotropic effects within seconds to minutes, as compared to the oral route, taking 30–90 minutes. At increased doses, increased sense of well-being, relaxation, and enhanced sensations occur [9]. Similarly, if buprenorphine is taken intranasally, it increases abuse potential, but not with buprenorphine/naloxone, since naloxone also exhibits an enhanced intranasal absorption [10]. It is hypothesized that since buprenorphine is a partial agonist and therefore it will not provide the substantial euphoric effect that heroin, being a pure agonist, will provide, the person is susceptible to taking cannabis [11].

It is speculated that cannabis disturbs vascular tone and this, by various mechanisms, leads to impaired blood supply to brain tissue [12]. Cannabis related angiopathy has also been linked with ischemic brain events in persons with chronic marijuana use [13, 14].

Renard and Gaillard reported a case of a hemorrhagic stroke along with imaging identified vasospasm [15]. It is possible that our patient suffered a similar event and that, fortunately, no hemorrhagic transformation of the infarction occurred. The drugs (cannabis and buprenorphine) could very well have had a synergistic effect on the development and evolution of the stroke. The presentation in our case report is most likely a drug combination related vasculopathy, which underscores the role of follow-up imaging to see if the vasospasm resolved or not. Unless serial scans are done, it is often difficult to differentiate an atherosclerotic stenosis or a subocclusive clot on one given imaging alone. The most interesting feature is the resolution of the vasospasm on our three sequential images, namely, CTA, MRA, and, finally, cerebral angiogram.

Cannabis and buprenorphine have only once been reported together to be the suspected cause of a brain bleed. This case report is the first to suggest a possible synergistic effect of buprenorphine (prescription drug) and cannabis (now becoming a legal street drug in many states) causing potentially morbid stroke. With the expectation that cannabis use will multiply ample folds in the coming years, it is important for healthcare experts to be prepared for its implications. The most important of which will be its potentially synergistic interactions with other drugs potentially ranging from buprenorphines or other opioids to over the counter ephedrine.
